# State-independent inhibition of the oncogenic Kv10.1 channel by desethylamiodarone, a comparison with amiodarone

**DOI:** 10.1007/s00424-023-02893-x

**Published:** 2023-12-08

**Authors:** F. Gomez-Lagunas, C. Barriga-Montoya, J. P. Pardo

**Affiliations:** 1 Department of Physiology, School of Medicine, Universidad Nacional Autónoma de México (UNAM), Mexico City, México; 2 Department of Biochemistry, School of Medicine, Universidad Nacional Autónoma de México (UNAM), Mexico City, México

**Keywords:** Potassium channels, Eag, Kv10.1, Antiarrhythmics, Cole-Moore shift, Cancer

## Abstract

Kv10.1 is a voltage-dependent K channel whose ectopic expression is associated with several human cancers. Additionally, Kv10.1 has structure–function properties which are not yet well understood. We are using drugs of clinical importance in an attempt to gain insight on the relationship between pharmacology and characteristic functional properties of this channel. Herein, we report the interaction of desethylamiodarone (desAd), the active metabolic product of the antiarrhythmic amiodarone with Kv10.1: desAd binds to both closed and open channels, with most inhibition taking place from the open state, with affinity ~ 5 times smaller than that of amiodarone. Current inhibition by desAd and amiodarone is not synergistic. Upon repolarization desAd becomes trapped in Kv10.1 and thereafter dissociates slowly from closed-and-blocked channels. The addition of the Cole-Moore shift plus desAd open-pore-block time courses yields an increasing phase on the steady-state inhibition curve (H∞) at hyperpolarized holding potentials. In contrast to amiodarone, desAd does not inhibit the Kv10.1 Cole-Moore shift, suggesting that a relevant hydrophobic interaction between amiodarone and Kv10.1 participates in the inhibition of the Cole-Moore shift, which is lost with desAd.

## Introduction

Kv10.1 (Eag1) is a human, K-selective, voltage-gated channel coded by the HCNH1 gene [29]. Kv10.1 is mainly expressed in neurons, and notably its ectopic expression has a clear, although not yet well understood, role in cancers (e.g., see [17, 21, 22]), which endows this channel with pharmacological and clinical interest. Additionally, Kv10.1 has structural/functional properties, some of them just recently described that are not yet well understood [13, 30, 32], making Kv10.1 a channel of major biophysical interest. In this work, we employed available drugs of clinical importance with the aim to gain insight on the relationship between pharmacology and functional characteristics of this channel.

The time course of Kv10.1 K^+^ current (I_K_) characteristically depends on the resting membrane potential (HP) from which channels are activated. Briefly, as the HP is hyperpolarized, I_K_ develops a progressive initial lag [6] and a progressively slower, markedly sigmoidal, time course. These two in-series effects of the HP are known as the Cole-Moore shift of Kv10.1 (e.g., [3]).

Previously, we reported that the antiarrhythmic drug amiodarone inhibits the Cole-Moore gating shift, as well as I_K_ of Kv10.1 [2]. This finding suggests that amiodarone binds both within the K^+^ conduction pathway of the pore, as well as in a structural element(s) (voltage-sensor module and/or intracellular domains) that participate in Kv10.1 voltage-dependent gating.

Amiodarone (hereafter also named as Ad) is metabolized by the liver, and its main metabolic product named desethylamiodarone is a pharmacologically active metabolite [7] that inhibits the cardiac I_Kr_ (HERG), I_K1_ (inward rectifier K channel), and sodium (Nav1.5) channels. Regarding HERG channels, desethylamiodarone (hereafter also referred to as desAd) was reported to inhibit K^+^ flow in a way contingent on channel gating, with affinity ~ 3 times lower than that of amiodarone and shifting the activation voltage dependence by − 9 mV [33]; similarly, desAd inhibits I_K1_ with a potency approximately half of that of Ad [4]. On the other hand, both Ad and desAd inhibit Nav1.5 channels at similar concentrations, without affecting the voltage dependence of activation [9, 20].

As indicated by its name, desethylamiodarone lacks an ethyl group present in amiodarone [7, 19], which makes it slightly smaller and less hydrophobic than Ad. Considering that desethylamiodarone is pharmacologically active, in this work, we extended our study of the interaction of Kv10.1 with amiodarone and related molecules, by reporting the interaction of desAd with this channel. Due to its similarity with Ad, our hypothesis was that DesAd would inhibit I_K_, and we were interested in determining whether it may exert a significant effect on the Kv10.1 Cole-Moore shift and whether it may inhibit I_K_ in a way qualitatively different to Ad.

## Methods

### Cell culture

HEK293 cells stably expressing Kv10.1 channels were kept in culture at 37 °C in a humidified, 5% CO_2_ atmosphere, in DEMEM/F12 media supplemented with 10% FBS and containing 300 µg/ml Zeocin. Experiments were conducted ~ 1 h after plating the cells on glass coverslips [10]. Kv10.1 stably expressing cells was kindly provided by Drs. Walter Stühmer and Luis Pardo.

### Electrophysiological recordings

Macroscopic currents were recorded with an Axopatch 1D amplifier (Axon Instruments). Currents were filtered with the built-in filter of the amplifier and sampled with a Digidata 1322A interface, at frequencies satisfying the Nyquist criteria. Electrodes were made of borosilicate glass (KIMAX-51) pulled to 1–1.5 MΩ resistance. Membrane capacitance and series resistance were compensated with the built-in circuits of the amplifier; 80–90% series resistance compensation was applied. Experiments were done at room temperature, as previously reported [10].

### Solutions

The standard extracellular solution contained (in mM) 5 KCl, 2 CaCl_2_, 157 NaCl, 10 HEPES-Na, and pH 7.2. In Fig. 6A, the external solution also contained 0.1 mM MgCl_2_, a [MgCl_2_] that did not affect any of the measurements reported. Higher [KCl]_o_ solutions were prepared by iso-osmolar replacement for NaCl. The internal solution contained (in mM) 90 KF, 30 KCl, 10 EGTA-K, 2 MgCl_2_, 10 HEPES-K, and pH 7.2 as reported [10]. Desethylamiodarone (Santa Cruz Biotechnology) and amiodarone (Sigma) were dissolved in DMSO. At the higher concentration applied (5 µM desAd), DMSO did not affect the channels (not shown).

### Data analysis

**Fig. 1:**
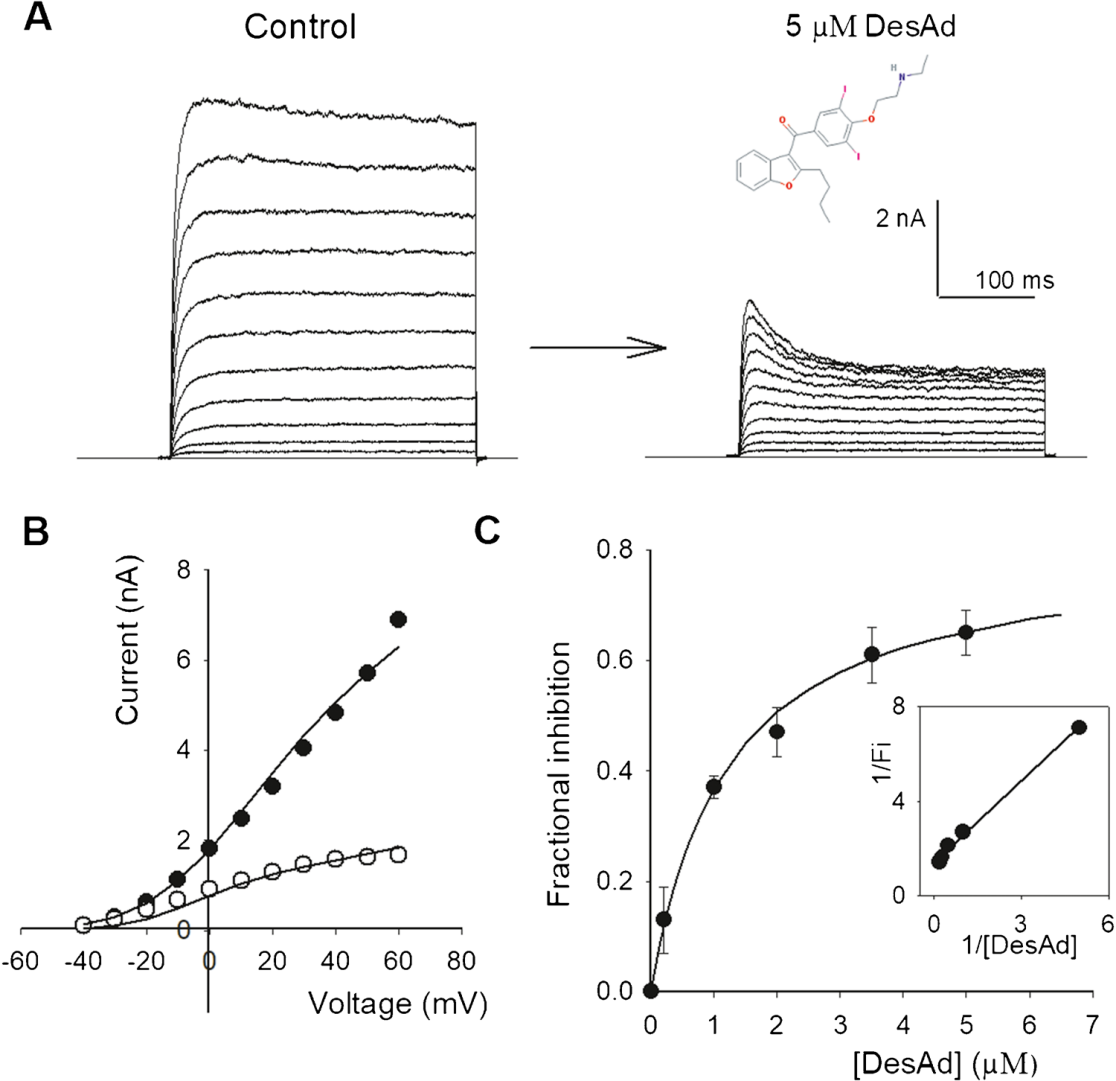
desAd inhibition of Kv10.1 channels. **A** K^+^ currents evoked by 250-ms pulses from −40 to + 60 mV applied in 10 mV steps, HP =  −70 mV. Left panel, control I_K_; right panel, I_K_ left after superfusing the cell with the extracellular solution containing 5 µM desAd. **B** Current (I_K_) vs. voltage (V_m_) relationship of the traces in A, I_K_ was assessed at pulse end. The lines are the least-squares fit of the points with Eq. 1 (see the “Methods” section) with parameters: control, *G*_max_ = 47.6 nS; *V*_1/2_ =  + 1.1 ± 1.5 mV, *z* = 2.2 ± 0.2; desAd, *G*_max_ = 13.2 nS, *V*_1/2_ =  − 12.9 ± 1.0 mV, *z* = 2.2 ± 0.2. *G*_max_ varies from cell to cell depending on the (highly variable) level of channel expression. Notice the left shift of the voltage dependence of activation (Δ*V*_1/2_ ~  −14 mV). **C** Dose–response relationship at + 30 mV (see Text). The line is the least-squares fit of the data with a Michaelis–Menten equation (Eq. 2, Methods) with maximal fractional inhibition FImax = 0.81 and Kd = 1.2 µM, obtained from the linearized double-reciprocal plot. The inset shows the double-reciprocal plot of the data. The straight line is the least-squares fit of the points with parameters: Kd = 1.2 µM and 1/FImax = 1.39. The linear relationship (*r* = 0.998) shows that desAd inhibits Kv10.1 following a Michaelis–Menten equation (with 1:1 stoichiometry)

Data in Fig. 1B was fit with a standard combination of Ohm and a two-state Boltzmann equations, namely:

1 $$I=\frac{{G}_{max}\left({V}_{m}-{V}_{k}\right)}{1-{e}^{\frac{-zF}{RT}\left({V}_{m}-{V}_{1/2}\right)}}$$

*G*_max_ is the maximal conductance; *V*_m_ is the pulse voltage; *V*_K_ stands for the K^+^ Nernst potential; *z* is the gating charge; *V*_1/2_ is the half activation voltage; *R*, *F*, and *T* have their usual meaning; and *T* was 298 K. Regarding the Boltzmann relationship embedded in Eq. 1, it should be mentioned that with a tetrameric channel with four identical, and independent, closed/open transitions, the activation curve should conceptually follow a 4th power Boltzmann distribution (e.g., [31]). Herein, in order to directly compare the new desethylamiodarone against the former amiodarone observations [2], a single Boltzmann distribution was used, which allows a direct comparison with our former observations, without invoking particular physical mechanisms which require further studies.

The dose–response data in Fig. 1C was fitted with a Michaelis–Menten equation:

2 $$FI=\frac{{FI}_{max}\left[desAd\right]}{{K}_{d}+\left[desAd\right]}$$

The line through the double-reciprocal points in Fig. 1C inset is the least-squares fit of the straight line:

3 $$\frac{1}{FI}=\left(\frac{{K}_{d}}{{FI}_{max}}\right)\frac{1}{\left[desAd\right]}+\frac{1}{{FI}_{max}}$$

FI stands for fractional inhibition, assessed as $FI=1-\frac{{I}_{desAd}}{{I}_{control}}$, where *I*control and *I*desAd are I_K_ at pulse end in control or desAd conditions, respectively; *K*_d_ is the apparent affinity of desAd for the channels; FI_max_ is the maximal fractional inhibition.

**Fig. 2:**
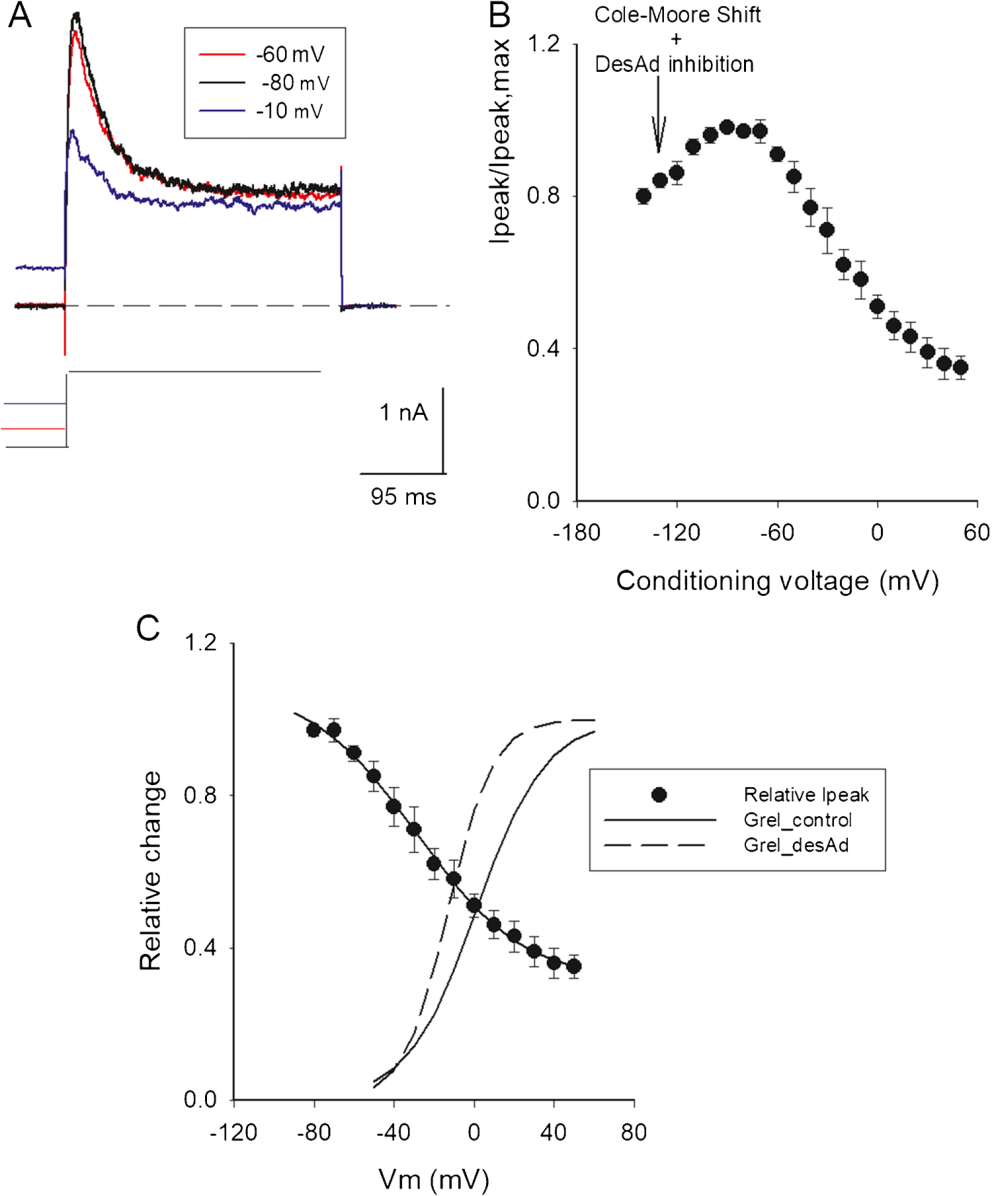
desAd steady-state inhibition in an extended voltage range. **A** Representative I_K_ recorded in the presence of 3.5 µM desAd. I_K_ was evoked by a + 50-mV/300-ms pulse applied immediately after 1-s conditioning pulses of the indicated voltages, applied from the HP of −70 mV, as indicated. **B** *I*_peak_/*I*_peak,max_ ratio at + 50 mV, as a function of the conditioning voltages. Note that peak I_K_ increases from −140 to approximately − 100 mV (see Text and the “Discussion” section). **C** Points in B re-plotted for conditioning voltages >  −100 mV, where I_K_ decreases monotonically with voltage. The line through the points is the least-squares fit of the data with a Boltzmann equation plus an offset (Methods), with parameters: *I*_peak,max_ = 0.8, *z* = 1.0, *V*_1/2_ =  − 28 mV, offset = C = 0.31. Notice that desAd binds (inhibits) the channels even before they open; to best see this, the figure shows normalized chord conductance vs. Vm curves of control (solid line, parameters: *z* = 1.5 ± 0.1, *V*_1/2_ =  + 1.1 ± 1.5 mV) and desAd blocked channels (dashed line, parameters: *z* = 2.2 ± 0.2, *V*_1/2_ =  −12 ± 1.0 mV), obtained from four experiments as in Fig. 1A; [desAd] = 3.5 µM

Data in Fig. 2C was fitted with a Boltzmann equation plus an offset:

4 $$\frac{{I}_{peak}}{{I}_{peak,max}}=\frac{1}{1+{e}^{\left(zF/RT\right)\left({V}_{cond}-{V}_{1/2}\right)}}+C$$

where *I*peak is peak I_K_ at + 50 mV, *I*_peak,max_ is the maximal peak I_K_, *z* is the apparent valence, *V*_cond_ is the conditioning voltage, *V*1/2 is the voltage at which (*I*_peak_/*I*_peak,max_) = 0.5, *C* is the offset, and *R*, *T*, *F* have their usual meaning.

### Test for competitive vs. non-competitive inhibition

We tested whether desAd (here for simplicity *D*_1_) and Ad (*D*_2_) inhibit I_K_ in a competitive (mutually exclusive) or non-competitive manner (Fig. 5).

For competitive inhibition (mutually exclusive), the total inhibition (FI_c_) is given by [25]:

5 $${FI}_{c}=1-\frac{1}{1+\frac{\left[{D}_{1}\right]}{{K}_{d1}}+\frac{\left[{D}_{2}\right]}{{K}_{d2}}}$$

where *K*_d1_ is *D*_1_ (desAd) affinity and *K*_d2_ is *D*_2_ (Ad) affinity (see Text).

Total non-competitive inhibition (FI_nc_) is given by [25]:

6 $${FI}_{nc}=1-\frac{1}{1+\frac{\left[{D}_{1}\right]}{{K}_{d1}}+\frac{\left[{D}_{2}\right]}{{K}_{d2}}+\frac{\left[{D}_{1}\right]\left[{D}_{2}\right]}{{K}_{d1}{K}_{d2}}}$$

If [*D*_1_] = *K*_d1_ and [*D*_2_] = *K*_d2_, Eq. 5 (competitive inhibition) predicts FI_C_ = 67%; Eq. 6 (non-competitive inhibition) predicts FI_nc_ = 75% (Fig. 6).

The competition plot of Cornish-Bowden [5] in Fig. 6 was done as previously reported [10] (see Text).

Results are the mean ± standard error of at least four independent experiments. Data analysis and curve fitting were carried out with Clampfit 10.6 (Axon Instruments), SigmaPlot 10, and GraphPad Prism 5.0. Kinetic schemes were simulated using SCoP (Simulation Resources Inc.). Statistical significance was assessed by *t*-test with cutoff *p* < 0.05, using GraphPad Prism software.

## Results

Compared to the Kv10.1 inhibitor amiodarone [2], its metabolic product desethylamiodarone lacks an ethyl group, which makes it smaller and less hydrophobic than amiodarone (Discussion). Figure 1A shows that despite this modification, desAd inhibits Kv10.1. The figure compares I_K_ elicited by −40 to + 60-mV pulses applied from the HP of −70 mV, before (control) and after superfusing the cell with an external solution containing 5 µM desAd, as indicated (see figure legend). See that desAD decreases I_K_ amplitude and at Vm ≥  + 10 mV, where I_K_ activation is comparatively fast, I_K_ presents a conspicuous decay phase due to drug entry into the open-pore from the cytoplasm, blocking I_K_ [1].

Kv10.1 inhibition by desAd is quantified in Fig. 1B, which presents the I_K_ vs. Vm relationship of the traces in A. The lines are the least-squares fit of the points with a standard equation that combines Ohm and Boltzmann equations (see the “Methods” section and Figure Legend). According to this, in addition to inhibit I_K_, desAd induces a ~  −14 mV shift to the voltage dependence of channels activation (*V*_1/2(control)_ =  + 1.1 ± 1.5 mV, *V*_1/2(desAd)_ =  −12.9 ± 1.0 mV), with a comparatively minor change of the apparent gating valence z (Δ*z*≈1.0) (see Fig. 2C and the “Discussion” section).

Figure 1C shows the concentration dependence of desAd inhibition at + 30 mV (a Vm at which channels open probability is ~ 20–25% away from its maximal value). The points are the average fractional I_K_ inhibition (FI) measured at pulse end (see figure legend and the “Methods” section). In order to avoid DMSO side effects on Kv10.1, the highest [desAd] tested was 5 µM (Methods). Note that at this [desAd], FI has not reached saturation; therefore, to best assess the dose–response curve parameters, the data was linearized as a double-reciprocal plot (Inset).

The least-squares straight line through the double-reciprocal points (correlation coefficient *r* = 0.998) indicates that desAd block follows a Michaelis–Menten saturation curve (line though the points of the direct plot) [25], with apparent affinity Kd = 1.1 µM. The latter is ~ 5 times smaller than Ad affinity (Kd = 0.2 µM) [2]. This affinity difference probably arises from a hydrophobic interaction between these molecules and its corresponding binding sites, which is weakened by the lack of an ethyl group in desAd (see the “Discussion” section).

### desAd inhibition under near steady-state conditions

In order to assess Kv10.1 inhibition under near steady-state conditions in an extended range of voltages, a standard two-pulse protocol was applied, consisting of long conditioning pre-pulses of varying amplitude, immediately followed by a constant test pulse to + 50 mV. The results are illustrated in Fig. 2A which compares three I_K_ recorded at the test pulse following 1-s conditioning pre-pulses of the indicated voltages, applied in the presence of desAd, as indicated (see figure legend).

Figure 2B presents the *I*_peak_/*I*_peak,max_ ratio vs. pre-pulse voltages relationship, of experiments as in A, where *I*_peak_ is peak I_K_ at + 50 mV following the indicated conditioning voltages and *I*_peak,max_ is the maximal peak I_K_. Notice that, peak I_K_ increases from −140 mV to approximately −100 mV, and thereafter decreases in a monotonical fashion as the conditioning voltage becomes more depolarized. The peak I_K_ increment seen at hyperpolarized voltages (−140 mV to approximately −100 mV) is the result of the addition of the Cole-Moore shift plus the time course of I_K_ inhibition (demonstrated in the “Discussion” section).

Therefore, in order to quantify desAd steady-state inhibition, the points in Fig. 2A were re-plotted within the window of −90 to + 50 mV (Fig. 2C). The line is the least-squares fit of the points with a monotonically decreasing Boltzmann equation added to an offset, with *V*_1/2_ =  −28 mV (see figure legend). Notice that, desAd inhibits Kv10.1 even at voltages at which channels do not conduct K^+^. The latter is seen more clearly by comparing the inhibition curve vs. the activation (Gk vs. Vm) curves of control and desAd-inhibited channels, as indicated (see figure legend). Note there is ~ 15–20% inhibition at voltages where channels are not conducting (Vm <  −50 mV). This shows that although desAd preferentially blocks open channels (as indicated by the decay phase of I_K_ [1]), it does not require Gk activation to bind to and to inhibit Kv10.1.

### desAd dissociation from closed Kv10.1 channels

**Fig. 3:**
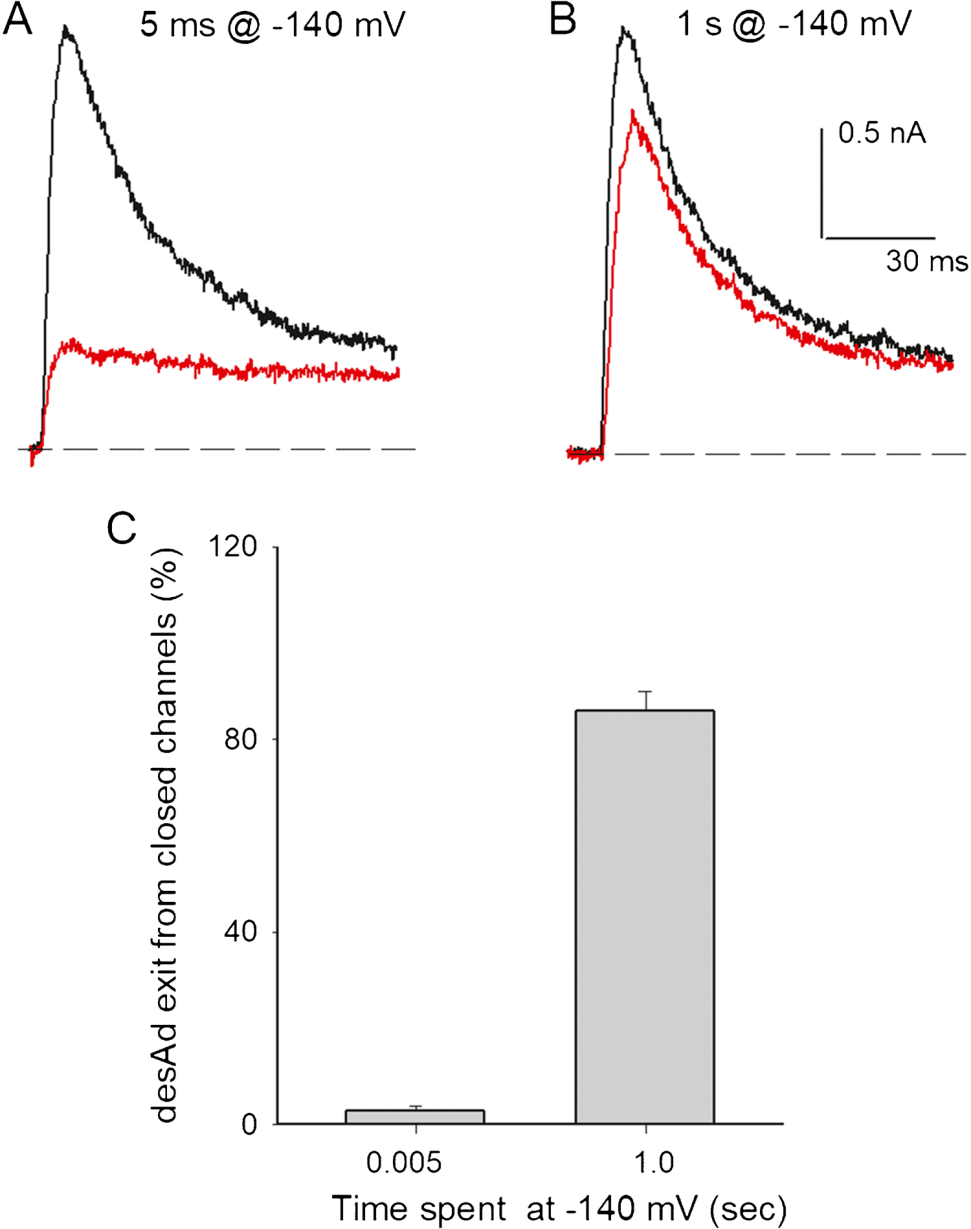
desAd dissociation from closed channels. **A** I_K_ evoked by a + 60-mV/120-ms conditioning pulse applied from − 70 mV, in the presence of 3.5 µM desAd (black trace), at pulse end, the membrane was repolarized to − 140 mV for 5 ms, and thereafter, I_K_ was elicited by a test + 60 mV pulse (red trace). Note that on the second pulse channels retained the level of block achieved at the end of the first pulse (see Text). This shows that desAd was trapped in the channels by the closing of the activation gate. **B** I_K_ recorded as in A, except that the time spent at − 140 mV, between activating pulses, was 1 s. There was ~ 80% recovery from blockage. This shows that desAd dissociates from closed channels. **C** Average extent of desAd leakage from closed channels (I_K_ recovery) after 5 vs. 1000 ms at −140 mV, *n* = 4

**Fig. 4:**
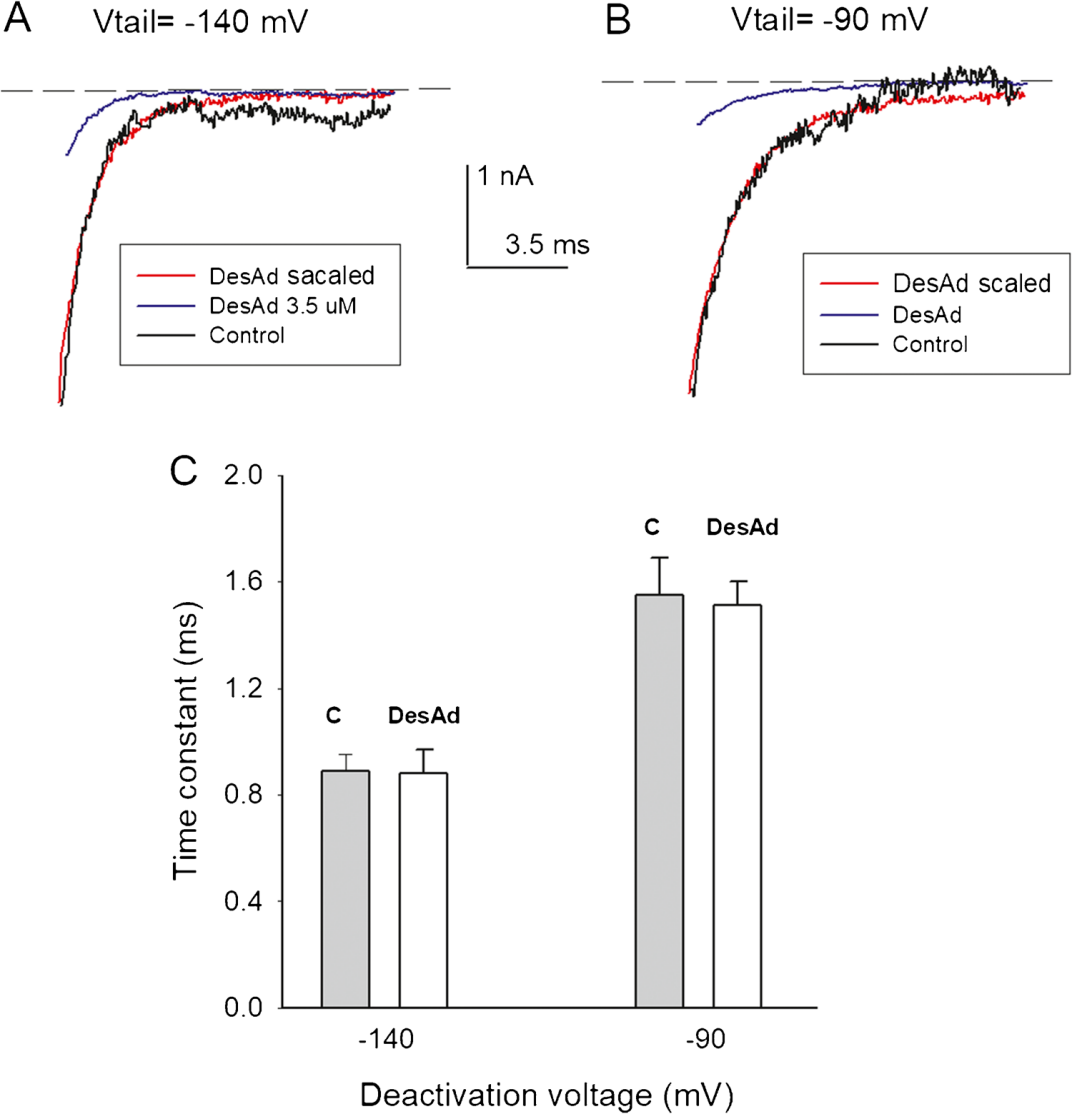
Deactivation tail currents of desAd-blocked vs. control channels. **A** Tail I_K_ at − 140 mV of control (black trace) vs. 3.5 µM desAd-blocked channels (blue trace). The red trace shows I_K_ of blocked channels scaled to match control I_K_ initial amplitude. **B** As in A but at the repolarization potential of −90 mV. Tail I_K_ was recorded following a + 30/100-ms activation pulse, applied from the HP of −70 mV (not shown in the figure). **C** Average deactivation time constants. Time constants are not significantly different (*p*(− 140 mV) = 0.98, *n* = 5; *p*(− 90 mV) = 0.93, *n* = 5)

Considering the above observations, we tested whether blocked channels could close trapping desAd within them. To assess this, we applied an experimental protocol similar to that previously used by Homgren et al. and Melishchuk and Armstrong [12, 16] studying Shaker channels blocked by TEA analogues, namely: a first + 60-mV pulse was delivered in the presence of 3.5 µM desAd to open and block the channels; then, the membrane was repolarized to − 140 mV to deactivate the channels fast (deactivation time constant ≈ 1 ms, see Fig. 4), and shortly thereafter (5 ms—a time intended to just allow full deactivation), a second + 60-mV pulse was applied to test the state of the channels (blocked vs. non-blocked). The representative traces in Fig. 3A show that I_K_ evoked by the second pulse (red trace) has an amplitude roughly equal to that at the end of I_K_ of the first pulse (i.e., equal to the non-blocked fraction of the first pulse, black trace) and that additionally it does not present the conspicuous decay phase of I_K_ of the first pulse. These observations show that most channels (~ 95%, Fig. 3C) closed trapping desAd within them [12, 16].

Thereafter, we tested an implication of the above observation, namely that desAd may dissociate from closed channels. In order to do that, we increase the time channels were kept at − 140 mV, from 5 ms to 1 s, before delivering the second activating pulse. Figure 3B illustrates the result of this experiment. Note that a substantial fraction of blocked channels recovered from block (~ 80%) while they were kept closed for 1 s at −140 mV, a voltage at which channels dwell in closed states far from the open state, and from which a rate-limiting step has to be overcome to reach closed states near the open state (e.g., [24, 26, 28]). The histogram in Fig. 3C quantifies these observations. The above shows that desAd block is reversible and that desAd dissociates slowly (as compared to the deactivation rate) from closed Kv10.1 channels.

desAd trapping upon closure of the activation gate is further demonstrated by comparing deactivation tail currents of control vs. desAd-blocked channels. Figure 4A, 4 illustrates tail I_K_ at the repolarization potentials of either −140 or −90 mV, respectively, recorded after activating the channels with a + 30 mV/100-ms pulse (not shown in the figure—see figure legend). As expected, tail I_K_ of blocked channels has a smaller amplitude than those of non-blocked channels. Hence, to compare their time course, tail I_K_ of blocked channels was scaled (red traces) to match the initial amplitude of control I_K_. Note that the time course of deactivation was not noticeably altered by desAd block, as quantified in the histogram in Fig. 4C (see the “Discussion” section).

### Cole-Moore shift of desethylamiodarone vs. amiodarone blocked channels

**Fig. 5:**
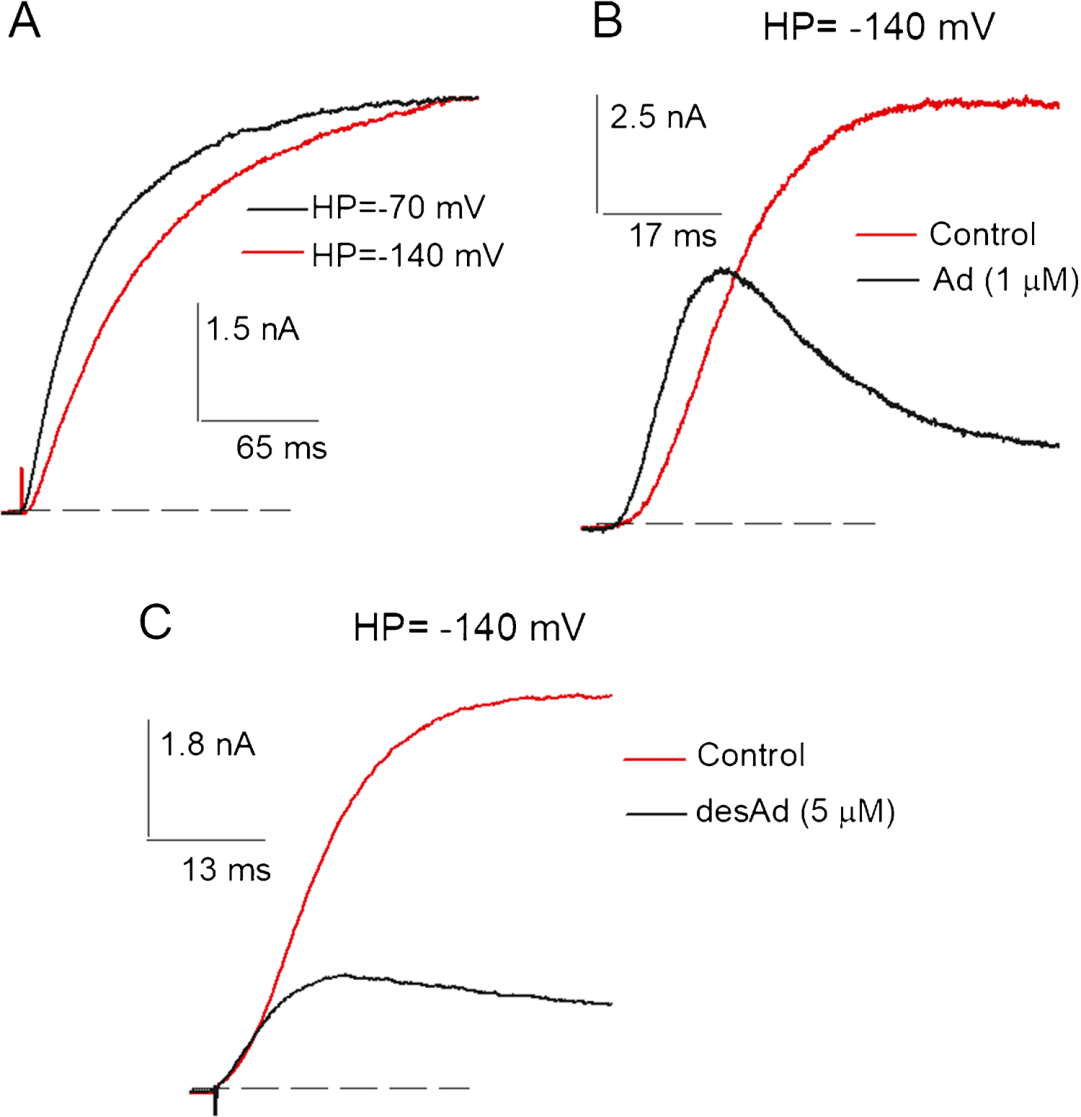
A test for desAd inhibition of the Cole-Moore shift. **A** Control I_K_ at + 30 mV evoked from either the HP of −140 or −70 mV, as indicated. Note the ~ single-exponential time course of I_K_ evoked from −70 mV and the slower, sigmoidal, time course of I_K_ evoked from −140 mV, characteristic of the Kv10.1 Cole-Moore shift. **B** I_K_ illustrating the previously reported Ad inhibition of the Cole-Moore shift. The panel shows superimposed traces of control (red) vs. Ad (1 µM)-blocked channels, as indicated. See that Ad inhibits the lag and the markedly sigmoidal time course of control I_K_ (see Text). **C** Superposed I_K_ recorded as in B, under control (red) and 3.5 µM desAd conditions, see that desAd does not inhibit the Cole-Moore shift (see Text)

Previously, we reported that amiodarone inhibits the Cole-Moore shift of Kv10.1 [2]. Thus, in order to advance our understanding of this inhibition, we tested whether the lack of an ethyl group in desAd could affect the drug capability to inhibit this process (Fig. 5).

For a reference, Fig. 5A illustrates the Cole-Moore shift of Kv10.1. The traces show I_K_ at + 30 mV evoked from either −140 or −70 mV, as indicated. Note the marked dependence of I_K_ time course on the HP. In particular, notice that I_K_ evoked from −70 mV approximately follows a single-exponential time course, while I_K_ evoked from −140 mV, in addition to an initial lag, exhibits a markedly sigmoidal time course, which characterize the Kv10.1 Cole-Moore shift (Discussion) (e.g., [3, 6, 24, 26, 28].

Also for a reference, Fig. 5B illustrates the previously reported Ad inhibition of the Kv10.1 Cole-Moore effect. The figure shows I_K_ at + 30 mV evoked from the HP of − 140 mV, of control vs. Ad-blocked channels as indicated. Notice that Ad decreases the lag and the slow, sigmoidal, time-course characteristic of the Kv10.1 Cole-Moore shift and that as a consequence, in spite of being a potent Kv10.1 blocker, I_K_ of Ad-modified channels is paradoxically initially bigger than control I_K_. This clearly demonstrates the inhibition of the Cole-Moore shift carried out by amiodarone (see [2, 10]). Also, notice that thereafter I_K_ in the presence of Ad becomes smaller than control I_K_ and develops a noticeably decay blocking phase, as expected.

In contrast to the latter, I_K_ in Fig. 5C illustrates that removal of an ethyl group of Ad renders the resulting desAd molecule unable to inhibit the Cole-Moore shift up to a 5 µM concentration: Note that the time course of I_K_ surge is never faster in desAd blocked than in control channels and that therefore I_K_ of desAd-blocked channels never surpasses control I_K_. The same result was obtained in all the cells tested (*n* > 5). This shows that desAd does not appreciably inhibit the Cole-Moore shift. The latter suggests that a significant hydrophobic interaction between amiodarone and Kv10.1 enables this molecule to inhibit the Cole-Moore shift with high affinity, an interaction that is lost with desethylamiodarone.

### A test for desethylamiodarone vs. amiodarone competition at inhibiting IK

**Fig. 6:**
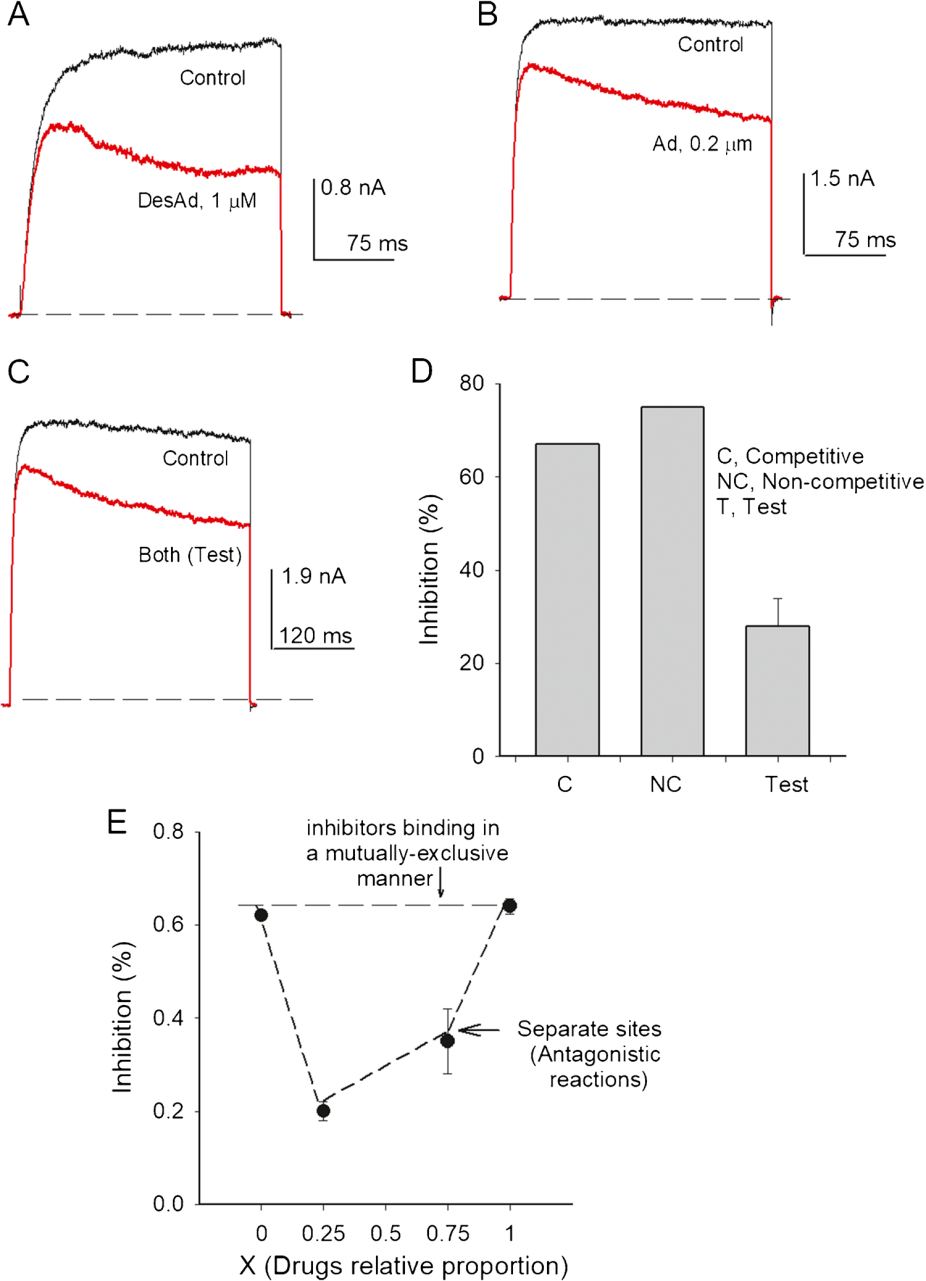
desAd and Ad inhibit I_K_ in an apparently non-competitive fashion. **A** Superposed traces illustrating I_K_ block by 1 µM desAd. I_K_ was evoked by a + 30-mV pulse applied from − 70 mV. **B** Traces illustrating I_K_ block by 0.2 µM Ad, recorded as in A. **C** desAd (1 µM) plus Ad (0.2 µM) joint inhibition of I_K_. **D** Comparison of the average extent of inhibition exerted by desAd plus Ad (Test) vs. the expected level of inhibition considering either a mutually exclusive (competitive, labeled C) or a non-competitive (NC) model of drugs interaction (see the “Methods” section). **E** Competition plot (see Text): the % Ik inhibition at + 30 mV was assessed as a function of the proportion (molar fraction) of desAd and Ad, as the concentration of each of them causing ~ 60% inhibition was reciprocally changed as X as: A*x*, B*x*-1, with *X* from 0 to 1

Considering the different drugs effect on the Cole-Moore shift, we were interested in testing whether Ad and desAd would inhibit I_K_ either in a mutually exclusive (competitive) or in a not mutually exclusive manner. To achieve this goal, we first assessed I_K_ inhibition exerted by either desAd or Ad alone, as well as when added together, with each drug added at its corresponding [K_d_] (see the “Methods” section). The results are illustrated by I_K_ in Fig. 6A–C which exemplify I_K_ block, as indicated.

The histogram in Fig. 6D compares the average I_K_ inhibition against the prediction of competitive (labeled c) vs. non-competitive (nc) inhibition models (see the “Methods” section). The non-competitive model assumes that both compounds can simultaneously bind to its target (enzyme/channel) in an independent way (i.e., the binding of one of them does not affect the binding of the other). Note that both models predict an inhibition larger than that empirically measured when both compounds are added simultaneously, with each of them added at its corresponding *K*_d_ concentrations.

Note that our results (bar labeled Test) show that I_K_ inhibition carried out by desAd and Ad: (1) is not synergistic, (2) exclude a mutually exclusive binding mechanism, and (3) also exclude a mechanism where both compounds can bind simultaneously in a non-interacting independent manner (non-competitive model). Therefore, to get a better insight regarding I_K_ inhibition by desAd in comparison to Ad, we performed the so-called inhibition plot of Cornish-Bowden [5, 10], an exhaustive test that allows to determine the way in which two inhibitors bind to its target (channel/enzyme) (see the “Methods” section).

To perform the inhibition plot two [drug] yielding, approximately same extent of inhibition (in this case ~ 60%) is selected (A, D: where A stands for Ad and D for DesAd); thereafter, the drugs are added together at a variable molar fraction X (A*x*, B*x*-1, with *x* from 0 to 1), and the extent of inhibition is tested. The Cornish-Bowden plot in Fig. 6E shows a clear downward deflection from the horizontal line connecting the pure Ad (*X* = 1) to the pure desAd inhibition points (*X* = 0). As indicated in the figure, this result excludes both the competitive binding mechanism and the non-competitive model (in agreement with Fig. 6D, see [5, 10]; instead, the plot indicates an antagonistic (not mutually exclusive) binding mechanism, which yields an inhibition smaller than that produced by each compound added alone (in this case ~ 60%) or by the addition of the two inhibitors (Fig. 6, NC). In any case, the presence of two binding sites for the two ligands is required for the emergence of the antagonistic binding mechanism (Cornish-Bowden).

## Discussion

The metabolic removal of an ethyl group from amiodarone yields the smaller and less hydrophobic molecule desethylamiodarone. An approximate comparison regarding desAd vs. Ad water solubility can be obtained with an empirically derived equation that accounts for the water solubility of a straight hydrocarbon, as a function of its carbon-chain length [14, 27]:

$$\mathrm{\mu o}(\mathrm{HC})-\mathrm{\mu o}(\mathrm{W})=-2436-884\times \mathrm{NC}$$

µo is the standard chemical potential, HC stands for a liquid hydrocarbon, W for water, and NC represents the number of carbon atoms of the chain.

According to this equation, removal of a two-carbon, ethyl group would change water solubility by:

$$\mathrm\Delta\mathrm\Delta\mathrm\mu\mathrm o\hspace{0.17em}\sim\hspace{0.17em}7248\;\mathrm{Joules}/\mathrm{mol}$$  

In the case of desAd vs. Ad block, this solubility change is accompanied by ~ fivefold affinity decrement of desAd as compared to Ad, that is a ΔΔG ~ 3626 Joules/mol, roughly half the energy change on water solubility.

Although not quantitatively precise, the above calculations suggest that a relevant hydrophobic interaction between Ad and Kv10.1 takes place in I_K_ inhibition. This possibility agrees with molecular dynamics simulations done on a homology model of the related HERG channel pore built against the KcsA pore, which suggested important hydrophobic interactions between pore blocking compounds, similar to Ad, and the intracellular pore region (see [8, 18]). On the other hand, the fivefold different affinity of these drugs could just simply be related to their binding to different sites, and hence, it may or may not be related to their different hydrophobicity.

Interestingly, and related to the latter, our observations indicate that in spite of their considerable different block affinities, both drugs shift the activation curve towards the left in a similar extent (Δ*V*1/2(desAd) =  −14 mV vs. Δ*V*1/2(Ad) =  −17 mV [2]), maybe this is because at the concentrations tested the corresponding locations where Δ*V*1/2 shift is exerted might be approximately equally saturated. On the other hand, Ad and desAd induced Δ*V*1/2 changes are comparable to the −9 mV shift induced by desAd on the related HERG channel [30]. Whatever the mechanism underlying the induced *V*1/2 changes, these observations suggest that drug-induced shifts of the activation curve are not straightforwardly related to the drugs I_K_ blockage affinities.

### Steady-state IK inhibition by desAd

The steady-state inhibition curve (Fig. 2A) shows an increasing phase (−140 to approximately −100 mV), prior to the monotonically decreasing phase seen at more depolarized conditioning voltages. The latter phase follows a monotonically decreasing Boltzmann equation (Fig. 2B) and shows that desAd is able to inhibit at voltages at which channels are closed (Vm <  −50 mV) (see below).

Regarding the increasing phase, it seemed to us that this phase was the result of the kinetic addition of pore block at the test potential (+ 50 mV) plus the channels Cole-Moore shift which is significant at hyperpolarized holding voltages (Vm ≤  −100 mV).

In order to qualitatively substantiate the above statement, we considered a simplified linear kinetic scheme for I_K_ at + 50 mV, with a single closed state C_near_, condensing the set of closed states near the open state, and a single C_far_ state representing the set of closed states far from the open state; C_far_ is connected to C_near_ by a rate-limiting transition. Channels dwell in C_far_ at hyperpolarized HPs (here −140 mV), and the rate-limiting passage from C_far_ to C_near_ upon depolarization yields the conspicuous lag and sigmoidal I_K_ time-course characteristics of the Kv10.1 Cole-Moore shift. On the other hand, at relatively depolarized HPs (here − 70 mV) channels dwell in C_near_ states; hence, I_K_ time course approaches a single-exponential time course, as reported (e.g., [23, 24, 26, 28]) (Fig. 5A).

**Fig. 7:**
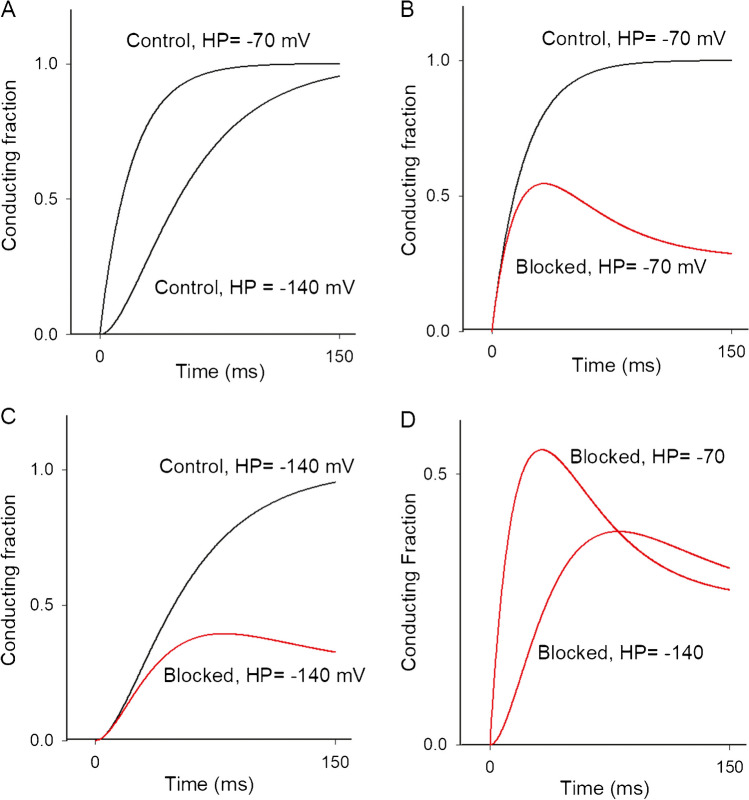
Qualitative I_K_ simulations. **A** Control I_K_ evoked by a + 50-mV pulse applied from either −70 or − 140 mV resting potentials, as indicated ([*B*] = 0, see Text). Note the Cole-Moore shift of I_K_ evoked from −140 mV. **B** Control ([*B*] = 0) vs. desAd-blocked ([*B*] = 3.5 µM) I_K_ at + 50 mV, both I_K_ activated from −70 mV (channels dwell in Cnear at the start of the pulse). Note the single-exponential activation time course. **C** Control ([*B*] = 0) vs. desAd-blocked I_K_ at + 50 mV, both I_K_ activated from −140 mV (channels dwell in C_far_ at the start of the pulse). Note the sigmoidal activation time course and the DesAd lack of inhibition of the Cole-Moore shift. **D** Comparison of desAd-blocked I_K_ evoked from either −70 or −140 mV, as indicated. Note that peak I_K_ is bigger, in an extent comparable to that seen in Fig. 2A, when channels are activated from −70 than when they are from −140 mV. This difference qualitatively explains the increasing phase of the steady-state inactivation curve. *K*_fast_ = 0.05 ms^−1^, *k*_slow_ = 0.025 ms^−1^, *K*_r_ = 0.008 ms^−1^, *K*_b_ = 0.022 (ms*µM)^−1^, [*B*] = [desAd] = 3.5 µM

Finally, since closed-state inhibition accounts for ~ 15% of total desAd blockage up to the conditioning voltage of −70 mV (Fig. 2B), we considered desAd block only from the open state:
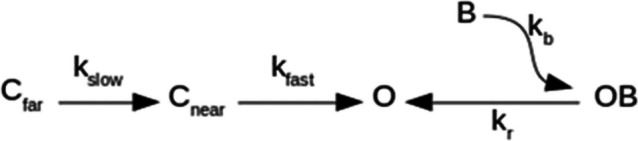


where *k*_fast_ is a first-order rate constant connecting C_near_ to the open sate O; *k*_slow_ is the slow (*k*s_low_ < *k*_fast_) rate-limiting constant that connects far (C_far_) to near (C_near_) closed states; *K*_b_ is the second-order rate block constant; *k*_r_ is a reverse rate constant, needed to provide steady-state inhibition at pulse end; B stands for blocker (desAd); and OB stands for open-and-blocked state (Fig. 7 legend).

Figure 7A compares simulated control I_K_ at + 50 mV evoked from either −70 or −140 mV, as indicated. Note the single-exponential character of I_K_ from − 70 mV, as well as the slower, markedly sigmoidal, time course of I_K_ evoked from −140 mV, which is the hallmark of the Kv10.1 Cole-Moore shift. Figure 7B shows simulated control I_K_ vs. I_K_ in the presence of desAd, both evoked from −70 mV. Similarly, Fig. 7C presents the corresponding simulated I_K_ evoked from − 140 mV, as indicated. Notice that, as observed (Fig. 5), desAd block does not inhibit the Cole-Moore shift (see figure legend).

Finally, Fig. 7D compares desAd block of I_K_ triggered from either −70 or −140 mV. Note that peak I_K_ (HP =  −70) > peak I_K_ (HP =  −140 mV), in an extent comparable to that empirically observed (Fig. 2B). This qualitatively explains the rising phase of the steady-state inhibition curve (Fig. 2A) at hyperpolarized voltages.

### desAd trapping upon closure of the activation gate

In agreement with the observation that desAd inhibits both open and closed channels (i.e., exerts a state-independent inhibition), we show that upon repolarization, most blocking desAd molecules become trapped within the channels (Fig. 3) and that thereafter dissociate slowly from the blocked-and-closed channels, attaining ~ 80% dissociation after 1 s at −140 mV. Supporting the latter, we also found that desAd does not interfere with Kv10.1 closing (Fig. 4). It is interesting to mention that none of the drugs that we have tested so far (mibefradil, amiodarone, dronedarone, desethylamiodarone, and quinidine) has hindered the activation gate closing. Blocker obstruction of pore closing may arise from relative drug vs. cavity sizes, or/and by the way in which a drug internalizes (binds) in the channels (e.g., see [12, 16]). Regarding the latter, it is interesting that in contrast to canonical Shaker-like K channels, the Kv10.1 pore, closed by Ca^2+^-calmodulin, does not show a conspicuous central cavity [30], so we would have expected that at least some of the drugs tested should have delayed gate closing. Hence, we hypothesize that the lack of drug effect on pore closure could be related to differences in the specific mechanism of activation gate closing, which seems different, and is less well understood, in Kv10.1 channels than in canonical K (Shaker-like) channels (e.g., see [11, 12, 33]). We consider that further work is needed to clarify these observations.

### Desethylamiodarone vs. amiodarone effects on the Cole-Moore shift

An interesting difference regarding desAd vs. Ad interaction with Kv10.1 is the lack of effect of the former on the Cole-Moore shift. We interpret this as suggesting that a significant hydrophobic interaction (lost in desAd) between Ad and Kv10.1 takes place in the inhibition the Cole-Moore shift. The latter supports previous observations showing that dronedarone, a related and also less hydrophobic molecule than Ad, in this case due to the addition of a hydrophilic group, is similarly unable to inhibit the Kv10.1 Cole-Moore shift [15]. On the other hand, we previously showed that mibefradil, a water-soluble molecule up to a ~ 35 mM concentration, inhibits the Kv10.1 Cole-Moore [10]. We hypothesize that mibefradil and amiodarone inhibit the Cole-Moore shift upon binding to different sites of the channels.

## Data Availability

Article data is available under reasonable request.
